# Persistent Periodic Uplink Scheduling Algorithm for Massive NB-IoT Devices

**DOI:** 10.3390/s22082875

**Published:** 2022-04-08

**Authors:** Tin-Yu Wu, Ren-Hung Hwang, Abhishek Vyas, Chia-Yiu Lin, Chi-Ruei Huang

**Affiliations:** 1 Management Information Systems Department, National Pingtung University of Science and Technology, Pingtung 912301, Taiwan; tyw@mail.npust.edu.tw; 2 Computer Science and Information Engineering Department, National Chung Cheng University, Chiayi 621301, Taiwan; vyas109p@cs.ccu.edu.tw (A.V.); ljy110p@cs.ccu.edu.tw (C.-Y.L.); pei4b066@gmail.com (C.-R.H.)

**Keywords:** 5G, NB-IoT, uplink scheduling, resource units, power saving mechanism, NPUSCH, massive IoT devices

## Abstract

Narrowband Internet of Things (NB-IoT) is one of the low-power wide-area network (LPWAN) technologies that aim to support enormous connections, featuring wide-area coverage, low power consumption, and low costs. NB-IoT could serve a massive number of IoT devices, but with very limited radio resources. Therefore, how to enable a massive number of IoT devices to transmit messages periodically, and with low latency, according to transmission requirements, has become the most crucial issue of NB-IoT. Moreover, IoT devices are designed to minimize power consumption so that the device battery can last for a long time. Similarly, the NB-IoT system must configure different power-saving mechanisms for different types of devices to prolong their battery lives. In this study, we propose a persistent periodic uplink scheduling algorithm (PPUSA) to assist a plethora of Internet of Things (IoT) devices in reporting their sensing data based on their sensing characteristics. PPUSA explicitly considers the power-saving mode and connection suspend/resume procedures to reduce the IoT device’s power consumption and processing overhead. PPUSA allocates uplink resource units to IoT devices systematically so that it can support the periodic–uplink transmission of a plethora of IoT devices while maintaining low transmission latency for bursty data. The simulation results show that PPUSA can support up to 600,000 IoT devices when the NB-IoT uplink utilization is 80%. In addition, it takes only one millisecond for the transmission of the bursty messages.

## 1. Introduction

Many Internet of Things (IoT) devices are available on the market; the so-called Internet of Everything (IoE) is becoming a trend [1,2]. Thanks to the availability of low cost, high speed, and highly reliable 5G cellular networks, people can purchase IoT devices and utilize them as per their needs [3,4,5,6]. However, different types of IoT devices have different transmission frequencies and reporting rates. Various telecom operators provide various tariff plans for their IoT device services. In narrow band Internet of Things (NB-IoT), the uplink data are transmitted through a narrowband physical uplink shared channel (NPUSCH), with limited transmission resources. Using Release 15 as an example, in reality, the highest uplink transmission rate is less than 200 kbps [7,8,9], and a large amount of flooded traffic at the same time will make NPUSCH very congested. Moreover, before a connection is established between user equipment (UE) and the evolved node-B or ’eNB’, a random-access procedure is required. After the random-access procedure is completed, all UE will need an appropriate uplink scheduling algorithm to transmit data to eNB with low latency in a power-saving mode (PSM). For this reason, we propose the persistent periodic uplink scheduling algorithm (PPUSA) to achieve energy-saving uplink scheduling.

With the use of the PPUSA algorithm, for UE, there can be a guarantee of uplink transmission periodically, without congestion in the uplink channel. The algorithm that is proposed in this paper will estimate the traffic demand of each type of UE to be uploaded to the base station based on the tariff flow and the data transmission characteristics of typical IoT devices. Generally speaking, the deployment of UE is carefully planned, so it will not transmit more than its subscribed traffic and will reserve a certain percentage of traffic for emergency use. The proposed PPUSA algorithm is designed to customize the upload schedule for the UE after taking into account the above considerations, which achieves power-saving and low-latency transmission and enables the entire NB-IoT system to cope with tens of thousands of uplink transmissions of IoT devices.

The novelty and contributions of this paper are summarized as follows. Firstly, this paper studies the NB-IoT uplink scheduling for a massive number of IoT devices, which receives less attention in the literature. Secondly, this paper proposes a novel uplink scheduling algorithm to solve the problem of transmitting messages with low latency in NB-IoT when there are many IoT devices, and each device demands that its sensing messages be transmitted in a periodic manner. In addition, each device may occasionally have emergency messages to send. Thirdly, the proposed novel scheduling mechanism guarantees that the device wakes up to transmit immediately in order to minimize transmission delay and power consumption. Finally, the proposed scheduling algorithm can support up to 600,000 IoT devices when the NB-IoT uplink utilization is 80%. In addition, it takes only one millisecond for the transmission of the emergency messages.

This paper is organized as follows. Section 2 describes the background information and related works, including the process of transmitting uplink data. Section 3 introduces and states the problem and how this paper attempts to solve it. Section 4 discusses the simulation results and conducts a data analysis. Finally, the paper concludes in Section 5 with a discussion of further enhancements of the work in future research.

## 2. Background and Related Works

### 2.1. About NB-IoT

NB-IoT was proposed by 3GPP to support a wide range of cellular devices and services. NB-IoT standard is one of the mainstream low power wide area network (LPWAN) technologies. The primary focus of NB-IoT involves its indoor coverage, long battery life, low cost, low power consumption, high connection density, and high throughput characteristics. NB-IoT has the unique feature to co-exist with 2G GSM systems, as well as with 4G LTE systems. Three operation modes, namely the in-band operation mode, guard-band operation mode, and stand-alone operation mode, respectively, are present in NB-IoT systems. Similar to LTE, NB-IoT adopts orthogonal frequency division multiple access (OFDMA) technology for downlink transmission with 15 kHz sub-carrier spacing. In uplink transmission, NB-IoT utilizes single-carrier frequency division multiple access (SC-FDMA) technology with 15 and 3.75 kHz spacing to support single-tone (sub-carrier) and multi-tone transmissions, respectively [10]. Table 1 depicts the technical parameters for the NB-IoT standard [11].

In NB-IoT, the following channels and signals are used in uplink transmission [12]:

1. Narrowband physical random access channel (NPRACH).
2. Narrowband physical uplink shared channel (NPUSCH).
3. Demodulation reference signal (DMRS).

Similarly, in NB-IoT, the following channels and signals are used in downlink transmissions [12]:

1. Narrowband physical downlink shared channel (NPDSCH).
2. Narrowband physical downlink control channel (NPDCCH).
3. Narrowband reference signal (NRS).
4. Narrowband primary synchronization signal (NPSS).
5. Narrowband secondary synchronization signal (NSSS).
6. Narrowband physical broadcast channel (NPBCH).

#### 2.1.1. Frame Structure for NB-IoT

**Figure 1:**
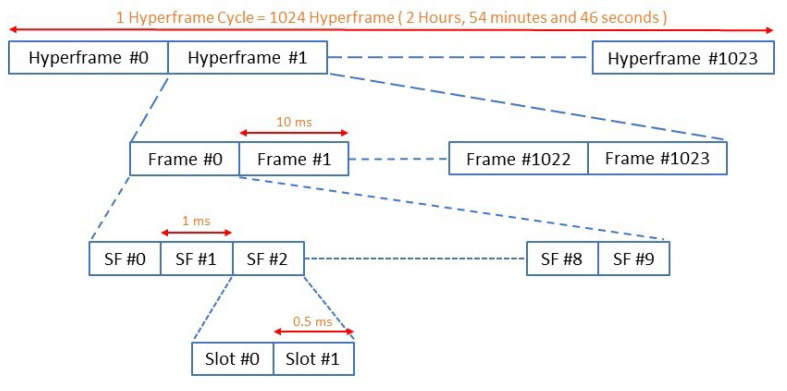
Frame structure—downlink and uplink sub-carrier spacing 15 KHz.

**Figure 2:**
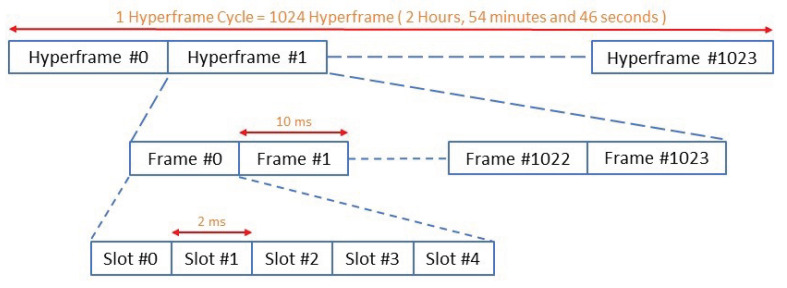
Frame structure—uplink with 3.75 kHz sub-carrier spacing.

The frame structure of NB-IoT is depicted in Figure 1 and Figure 2. The highest level starts with a hyperframe cycle, where one hyperframe cycle consists of 1024 hyperframes and each hyperframe has 1024 frames [12,13].

One frame is composed of ten subframes, and each subframe is divided into two slots each of 0.5 ms, which is similar to traditional LTE systems. In the downlink and uplink transmission, NB-IoT supports a sub-carrier spacing of 15 kHz, for which each frame consists of twenty slots. For the uplink, NB-IoT supports an additional sub-carrier spacing of 3.75 kHz. To accommodate this sub-carrier spacing, each frame is directly divided into five slots, each of two milliseconds [12,13].

#### 2.1.2. About Coverage Enhancement Level—CE Level

The 3GPP classifies coverage levels into three types, namely, CE Level 0, CE Level 1, and CE Level 2, respectively. There are certain differences in user equipment access and transmission mechanisms under these three coverage levels. The base station (BS) will divide the coverage into three levels based on the defined reference signal received power (RSRP) thresholds. Then the coverage is carried through the system information block-narrow band (SIB-NB). SIB-NB carries radio-resource control information to inform the user devices about the configuration of the NB-IoT physical random access channel (NPRACH) that contains three CE levels. The user’s device will find out which CE level it belongs to based on the measured RSRP and use the corresponding NPRACH configuration to carry out the random access procedure [14].

The coverage target of NB-IoT is ’maximum coupling loss’ (MCL) 164 dB. The coverage improvement is mainly achieved through repetition to ensure reliable connectivity in remote areas, basements, and other places with poor signal quality [10].

#### 2.1.3. Power Saving Mechanism of NB-IoT

**Figure 3:**
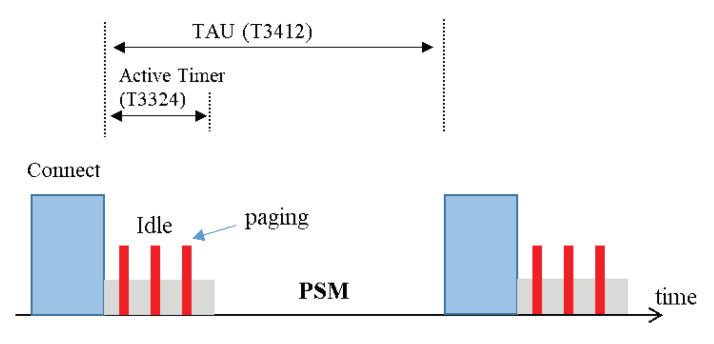
PSM-MODE.

**Figure 4:**
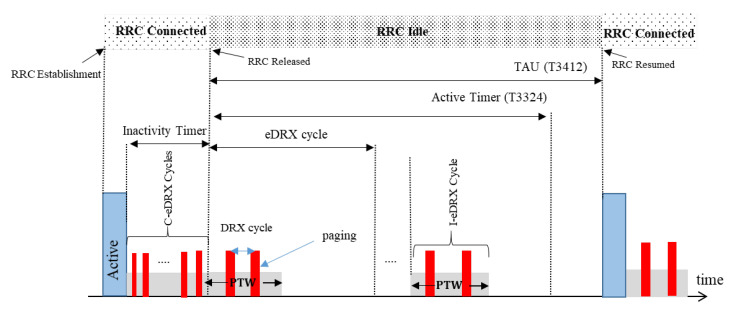
eDRX-MODE.

One of the main features of the 3GPP Release 14 of the NB-IoT is its long-term power-saving mode. Two different power saving mechanisms are defined for NB-IoT: (i) power saving mode (PSM) and (ii) extended discontinuous reception (eDRX). PSM was introduced in the 3GPP Release 12 [15]. Figure 3 shows the operating procedure of the PSM mode. Similarly, Figure 4 shows the operating procedure of the eDRX mode for the NB-IoT devices.

### 2.2. Uplink Scheduling for NB-IoT

When the base station wants to schedule an uplink transmission of the user equipment (UE), the base station sends a downlink control information (DCI) to one of the NPDCCH search spaces monitored by the UE. In order to distinguish the UE, the system assigns different radio network temporary identifiers (RNTI) to different UE, and the base station uses these RNTIs to encode the CRC bits of the DCI. Therefore, only the UE that knows the corresponding RNTI can successfully decode, and after decoding, the relevant information about the uplink schedule can become available.

**Figure 5:**
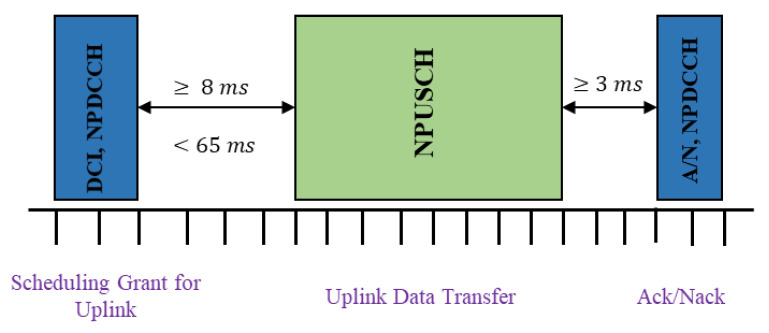
Uplink data transmission.

There are many types of DCI, among which, the DCI-format-N0 is used for uplink scheduling [16,17]. For uplink data transmission, the UE needs a time gap of at least 8 milliseconds to switch from receiving the DCI mode to the transmission mode. This time gap, also called scheduling delay, is designed for the UE to decode DCI. After the UE completes the NPUSCH uplink transmission, a time gap of at least 3 milliseconds is required to allow the UE to switch from the transmission mode to the receiving DCI mode to receive the ACK/NACK, and monitor the next NPDCCH search space, as shown in Figure 5.

### 2.3. Literature Review of Uplink Scheduling

The NB-IoT system supports single-tone (3.75 kHz) transmission and multi-tone (15 kHz) transmission for uplink scheduling. NB-IoT can be deployed in-band, utilizing resource blocks within a regular LTE carrier, in the unused resource blocks within an LTE carrier’s guard-band, or standalone for deployments in a dedicated spectrum. However, we cannot directly apply the LTE uplink scheduling mechanism to NB-IoT because the LTE’s semi-persistent scheduling (SPS) [18] is dedicated to periodic data transmissions, such as voice over IP (VoIP) services. Therefore, the existing LTE SPS configuration is only for a single UE. Moreover, in [19], the authors propose a group-based uplink scheduling algorithm, where they group the devices first and then select a group leader in each group. Next, the base station determines which group transmits first according to the environment of each group leader. However, this approach only schedules a single device.

In [20], the authors design a scheduler to arrange wireless resources for NB-IoT resource allocation. However, the scheduler is designed to comply with 3GPP Release 13 and cannot support single-tone and multi-tone transmissions. The work [21] proposes an adjustable uplink resource scheduling scheme, but the scheme only addresses scheduling for emergency needs and does not meet the NB-IoT specifications. In [22], the authors analyzed a single UE based on the queuing theory and used the analysis results to optimize the parameter configuration (such as retransmission times, scheduling delay) in NPRACH and NPDCCH. However, the work does not analyze NPUSCH. Other works in the literature can be divided into two categories: uplink scheduling for single-tone transmission [15,16,23] and uplink scheduling for multi-tone transmission [14,17,24]. This paper focuses on the second category. The main goal of this work is to schedule uplink transmission for as many UE as possible while maintaining a low transmission delay for emergency messages and high energy savings. To achieve this goal, we divide messages into periodic and emergency (referred to as “bursty” hereafter), and NB-IoT UE into three types, according to the time cycle of their periodic message reporting. Moreover, we also consider power saving mechanisms (PSM) and UE at the CE-level.

In the literature, studies only schedule massive numbers of UE of the same type and do not consider the two crucial power saving mechanisms, namely PSM and eDRX, respectively. Table 2 displays the comparison of related works.

### 2.4. Some Advances in NB-IoT

In NB-IoT, the maximum level of power consumption happens during the active time; that is, during Tx and Rx. In Release 16, it is expected that the UE will transmit when radio resource control (RRC) is in an ideal mode through Msg3 (RRC connection request) without the use of access grant. It is possible for a UE in an RRC connection mode to transmit data without a grant or using the simplified ’control-less’ grant. Yet another development is made in reducing the signaling overhead in NB-IoT without compromising the quality of service. The aforementioned features reduce both power consumption and latency. Release 16 also proposed further studying the original signal waveforms, such as FDMA, which require less orthogonality with more relaxed timing advance (TA) when compared to single-carrier frequency-division multiple access (SC-FDMA) [12].

In [25], based on the concept of grant-free communications, the authors investigated the adaptive period of the industrial Internet of Things (IIoT), where only some of the devices were active at a given time slot. The authors proposed two new schemes, namely, periodic block orthogonal matching pursuit (PBOMP) and periodic block sparse Bayesian learning (PBSBL). Both schemes outperform the previous schemes in factors such as the success rate of user activity detection (UAD), bit error rate, and accuracy in period estimation and channel estimation [25].

## 3. Proposed Uplink Scheduling Mechanism for NB-IoT

This section discusses the proposed uplink scheduling algorithm for NB-IoT systems and describes how to apply the power-saving mechanisms in the proposed system. In this paper, the UE are divided into three types based on [26,27], namely heavy, normal, and light types, respectively. Each type has different requirements, such as the reporting period, transmission rate, time slot, and power-saving parameters. Next, based on the algorithm proposed in [28], we designed an uplink scheduling algorithm to meet our requirements. UE, besides sending periodical reports, may send bursty reports. Therefore, according to their delay tolerances, UE data transmissions can include two types (see Table 3).

The NB-IoT was originally designed to serve many connections, and the system does not have strict delay requirements for UE. However, due to a wide variety of UE, when multiple connections coexist, bursty data from alarm systems and medical equipment may be blocked by periodic reports. Therefore, this paper classifies the types of UE and services. By using the proposed uplink scheduling algorithm, UE can transmit bursty messages without affecting periodical data transmission.

In the following section, we introduce the proposed PPUSA scheduling algorithm and the application of the power-saving mechanism (PSM). In the end, an overall system overview that integrates all of the proposed designs is discussed. The system parameters used are defined in Table 4.

### 3.1. Scheduling Algorithm (PPUSA)

In the proposed approach, the eNB uses PPUSA to schedule each type of UE. UE are activated at the specified time and follow the instructions of the eNB. UE know how many RUs they need to occupy, the time required for uplink transmission, and when to go into deactivation mode based on the downlink control information (DCI). Using the PPUSA, we attempted to achieve a massive number of connections, low-latency bursty transmissions, and low power consumption. In PPUSA, each type of UE has its report period. For example the UE report period from short to long is:

(1) $$T_{Heavy}<T_{Normal}<T_{Light}$$

In our proposed design, 20% of RUs of each frame (a scheduling period) are reserved for bursty message transmissions, i.e., each frame has 10 subframes (1.0 ms), but the fifth and tenth subframes are not scheduled for periodic type UE. Next, the scheduling orders for UE are heavy type, normal type, and finally light type. Heavy type UE have the shortest report period and, therefore, are allowed to schedule first. The principle of scheduling is not to occupy the same slots of previous scheduled UE. The overall process is presented below:

- Step 1: given *T* slots, the algorithm first marks each frame’s fifth and tenth subframes for bursty UE. As a result, there is an ordered set *S* of available slots for periodic UE that does not include slots occupied for bursty UE.
  (2) $$S=(S_{1},S_{2},S_{3}....).$$
- Step 2: schedule the heavy-type UE into set *S* in order. The UE is scheduled into the next empty slot according to its report period. In other words, for the UE of this type, during its report period, the first available time slot is reserved for this UE’s periodic report transmission. It is known as greedy scheduling in real-time scheduling.
- Step 3: schedule the normal type UE into set *S*, as in step 2. Similar to step 2, the schedule is based on the UE’s report period, and if the desired slot has been already occupied, the next first available time slot is reserved.
- Step 4: schedule the light type UE in the same way as the above steps.

Algorithm 1 describes the procedure of the PPUSA mechanism. The process of a new arrival UE is described in Algorithm 2.
**Algorithm 1** Persistent periodic uplink scheduling algorithm (PPUSA).**Input:***T*, $T_{Heavy}$, $T_{Normal}$, $T_{Light}$, $N_{Heavy}$, $N_{Normal}$, $N_{Light}$, $RU_{a}$**Output:***S*1:*S*:= a sequence of empty slots indexed 0 through *T*2:**for***j* = 1 to *T* **do**3: **if** *j* mod 5 = 0 **then**4:  $S_{j}$ is marked as reserve for bursty UE(R)5: **else**6:  $S_{j}$ is marked as empty7: **end if**8:**end for**9:**for***a*ϵ (Heavy, Normal, Light) **do**10: $N_{a}$ is the number of UE of type a11: $T_{a}$ is the reporting period of type a12: **for** *i* = 1 to $N_{a}$ **do**13:  // Schedule time slots for UE $a_{i}$14:  ;**repeat**15:  **for** *j* = 1 to $RU_{a}$ **do**16:   k= next smallest index of an empty slot from set S(k=1..T) //*k* = 0 initially17:   **if** *k* does not exist **then**18:    **return** scheduled failed19:   **end if**20:   $S_{k}$ = $a_{i}$21:  **end for**22:  $k=k+T_{a}$23:  **until** k≥T24: **end for**25:**end for**26:**return***S*

**Algorithm 2** Arrival of new UE.**Input:***T*, $RU_{New}$, $T_{New}$
**Output:**
*S*
1:**if** a new UE arrives **then**2: identify arriving UE’s CE level and type3: schedule from current time slot4: **repeat**5: **for** *j* = 1 to $RU_{New}$ **do**6:  *k* = next smallest index of an empty slot for set S(k+1..T) //*k* = (current time slot − 1) initially7:  **if** *k* does not exist **then**8:   **return** scheduled failed9:  **end if**10:  $S_{k}=a_{i}$11: **end for**12: $k=k+T_{New}$13: **until** k≥T14:
**end if**
15:
**return** *S*



### 3.2. Power Saving Mechanism (PSM)

According to [14], NB-IoT only supports the open loop power control in the uplink to achieve low complexity. Open-loop means UE can determine uplink transmission power according to MCS and RU, but eNB cannot send a power control command to UE to set its uplink transmission power. The UE transmit power $P_{NPUSCH,C}$(*i*) for NPUSCH transmission in the NB-IoT uplink slot *i* for serving cell *c*, given by [14]; if the number of repetitions of the allocated NPUSCH RUs is less than 2, then,

$$\begin{array}{c} P_{NPUSCH,c}\left(i\right)=min\{\left(P_{CMAX,c}\right),10log\left(M_{NPUSCH,c}\left(i\right)\right)+\\ P_{O-NPUSCH,c}\left(j\right)+\alpha_{c}\left(j\right)\cdot PL_{c}\} \end{array}$$

Otherwise,

(3) $$P_{NPUSCH,c}\left(i\right)=P_{CMAX,c}{\left(i\right)}_{\left[dBm\right]}$$

where $P_{CMAX,c}$(*i*) is the configured UE transmit power in the NB-IoT uplink slot *i* for serving cell *c*, $M_{NPUSCH,c}$(*i*) values are {1/4, 1, 3, 6, 12} and $P_{O-NPUSCH,c}$(*j*) is the sum of $P_{O-NOMINAL-NPUSCH,c)}$(*j*) and $P_{O-UE-NPUSCH,c}$(*j*), $\alpha_{C}$(*j*) = 1; for NPUSCH format 1, $\alpha_{C}$(*j*) is provided by higher layers for serving cell *c*. $PL_{c}$ is the downlink path loss estimate calculated in the UE for serving cell *c* in dB and $PL_{c}$ = nrs-Power + nrs-PowerOffsetNonAnchor − NRSRP, where nrs-Power is set according to the number of NRS antennas and nrs-powerOffsetNonAnchor is set to zero in our proposed approach.

In terms of power-saving mechanisms, previous works, including [29,30,31,32,33,34], have proposed different solutions. Users can change the PSM and eDRX timers on UE. In our proposed approach, UE do not need to receive signals from eNB after data transmission and will enter PSM directly once the data transmission is complete.

The average daily power consumption $E_{day_{Wh}}$ and battery life are calculated as follows:

(4) $$E_{day_{Wh}}=\frac{N_{reports_{day}}\times \left(E_{COM}+E_{IDLE}+E_{SLEEP}\right)}{3600}$$

(5) $$Y=\frac{C_{bat}}{E_{day_{Wh}}\times 365.25}$$

where $N_{reports_{day}}$ denotes the number of uplink reports sent in one day, $E_{COM}$ and $E_{IDLE}$ and $E_{SLEEP}$ denote the power consumption of the uplink transmission, eDRX, PSM, respectively, and $C_{bat}$ is the battery capacity (Wh).

**Figure 6:**
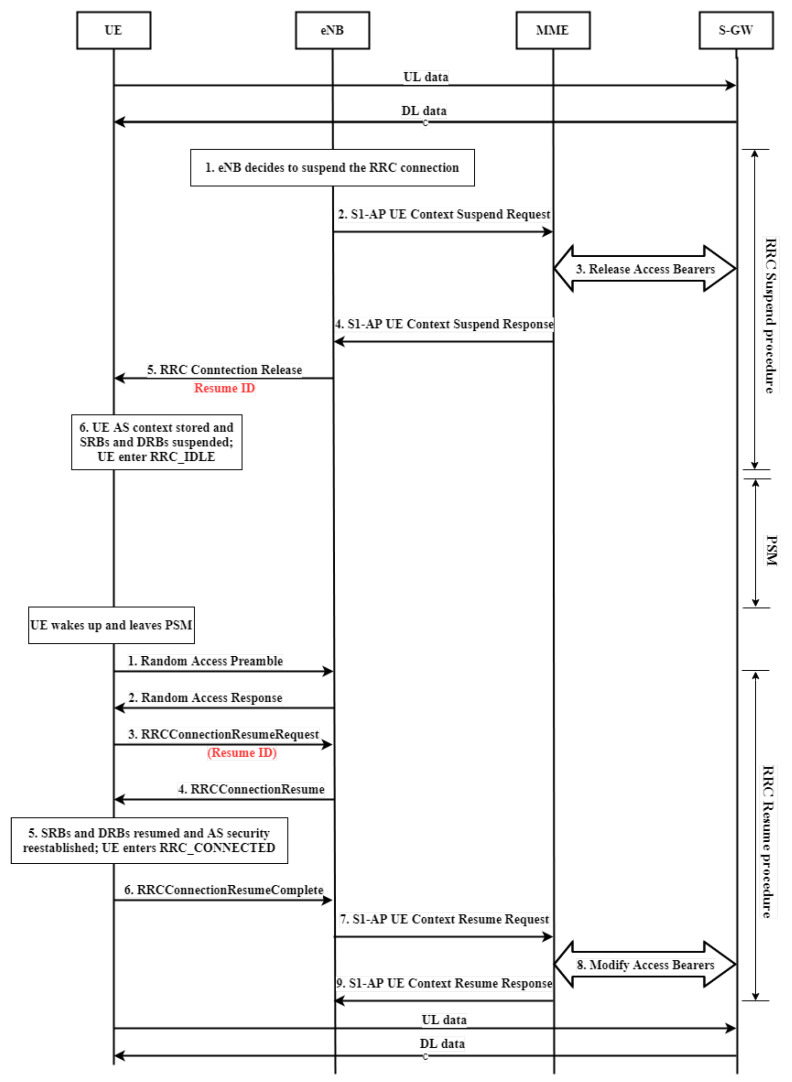
RRC resume and RRC suspend procedure.

In user plane optimization [35], NB-IoT introduces two new RRC states: RRC suspend state and RRC resume state, in order to reduce the number of messages that the NB-IoT UE needs to exchange when switching between the RRC connected mode and the idle mode, as well as to save power consumption, as shown in Figure 6. The eNB issues an instruction to make the UE enter the RRC suspend state when the UE leave the RRC connection, and the suspend instruction will carry a group of “Resume ID”.

Instead of converting the RRC connected mode to the idle mode, the eNB and UE retain most of the radio resources and security configurations used in the RRC connection mode. When the NB-IoT UE wants to transmit data, it only encloses the Resume ID allocated by the eNB in “message 3: RRC connection resume request” in the random access procedure. The eNB can identify the NB-IoT UE through this ’Resume ID’, skip the exchange of related configuration messages, and transmit data directly.

### 3.3. System Overview

**Figure 7:**
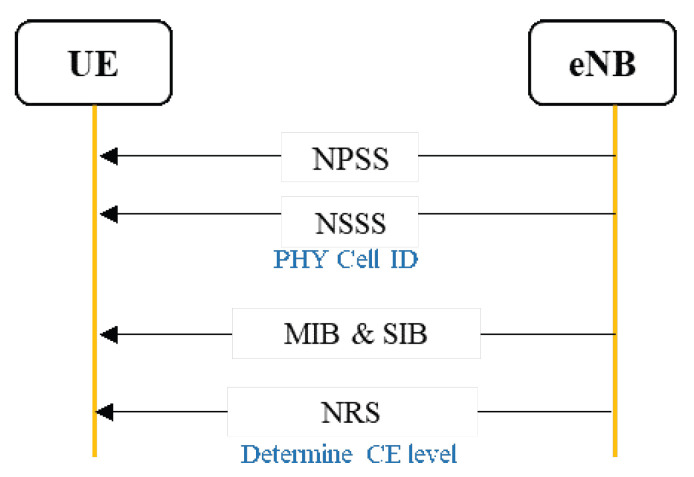
Synchronization and reading system information flow.

**Step 1: initial synchronization:** before the periodic uplink data transmission begins, the UE needs to read the narrow band primary synchronization signal (NPSS) and narrow band secondary synchronization signal (NSSS) broadcasted by the eNB on the anchor carrier to complete the synchronization. The physical cell ID is decoded from NSSS. In the next step, the UE reads the NB-IoT’s exclusive master information block-narrow band (MIB-NB) and system information block-narrow band (SIB-NB) broadcasted by the eNB on the anchor carrier to achieve synchronization, as shown in Figure 7.

MIB indicates the operating mode of the carrier and some scheduling information of the system information block-1 (SIB-1). It can be in-band, guard-band, or stand-alone. After reading the MIB, the UE will calculate the complete scheduling information by combining the SIB-1 scheduling information with previous physical cell identification. Using the same method SIB-1 will further contain the scheduling information of the other NB-IoT dedicated system information blocks (SIB-2-NB to SIB-16-NB). The UE must first read the NPRACH resource configuration information in SIB-2 before proceeding with the subsequent random access procedure.

**Figure 8:**
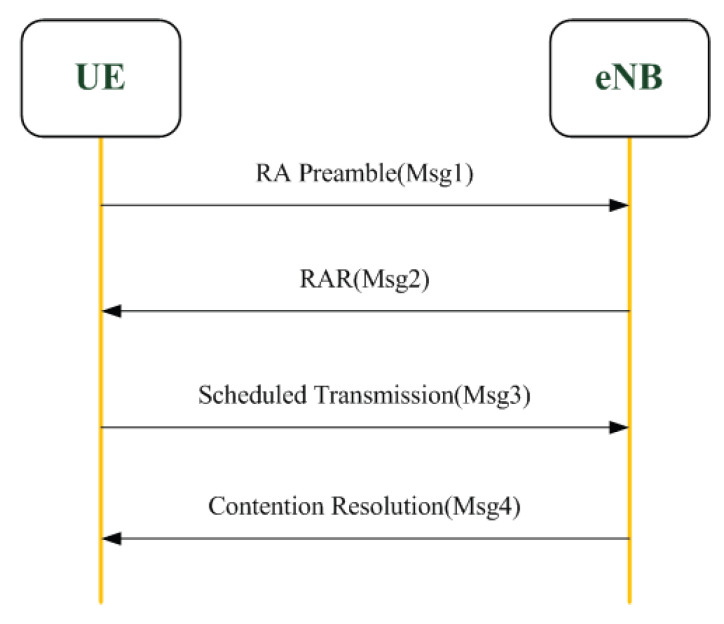
Random access procedure.

**Step 2: random access procedure:** in the NB-IoT network, devices need to perform a random access (RA) procedure to communicate with an eNB before data transmission. There are two types of RA procedures: connection-based and connection-free. The distinction is that the RA procedure is initiated by devices or by an eNB. The connection-based RA procedure is initiated by devices and is used when the devices have network access demands. There are four steps in the connection-based RA procedure, as shown in Figure 8.

**Figure 9:**
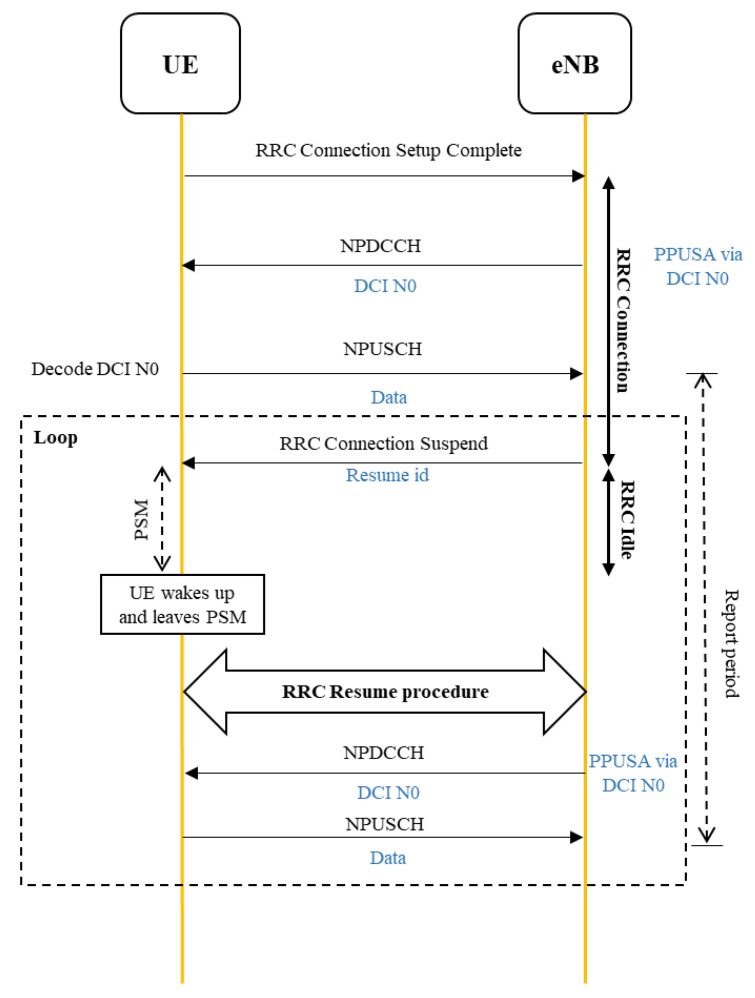
PPUSA procedure.

**Step 3: RRC connection setup complete:** UE returns *RRC connection setup complete* to eNB after the UE receive RRC an connection setup, as shown in Figure 9. Then the eNB transmits DCI through the NPDCCH, which carries the PPUSA scheduling strategy to the UE. After decoding DCI, the UE knows the scheduling information and transmits the uplink data to the eNB.

**Step 4: RRC connection suspend:** eNB directly starts the ‘RRC connection suspend’ procedure to switch the UE to the RRC idle state, and the suspend instruction will carry a group of ‘Resume ID’ after the UE transmits the data.

**Step 5: PSM:** the UE enters the PSM.

**Step 6: RRC resume procedure:** before the UE’s report period is expired, the UE leaves the PSM according to the sleep time. Then, the UE executes the RRC resume procedure and sends the resume ID to the eNB through Msg3. The eNB is able to identify the UE through the ’Resume ID’ and allows the UE to perform uplink data transmission. After that, the UE continues to loop the marked part in Figure 6 until the end of the scheduled cycle time.

## 4. Simulation Environment and Results Analysis

This section presents a simulation study of PPUSA. The simulator is a time-driven simulator written in Python language. We wrote the simulator according to the frame structure and all kinds of control and data channels specified in the 3GPP Release 13 and -14 standards [36,37], as described in Section 2.

### 4.1. Basic Parameters

As shown in Table 5, according to the efficiency, the amount of data that one RU can carry varies in NB-IoT uplink. In our proposed approach, we used the 12 tones of a multi-tone transmission.

### 4.2. Simulation Environment

Table 6 displays information about the simulation scenario. According to the specification of NB-IoT, the number of UE is more than 10,000 at each CE level. It is assumed that 1% to 10% of the UE are randomly selected to have bursty messages to send in each frame. The number of required RUs for sending bursty messages is set to either 1 or 2.

Table 7 displays the simulation parameters of three different simulation scenarios. According to [26], six representative types of UE are selected and the required RUs are calculated based on the three CE levels.

Table 8 displays the settings of powers parameters at each CE-level.

### 4.3. Performance Metrics

The following performance metrics are selected to evaluate the PPUSA’s capacity of the periodic and bursty devices and the performances are compared with [24].

- Average access delay.
- Number of serving NB-IoT UE and uplink resource utilization of the three CE levels.
- UE’s battery lifetime.

### 4.4. Simulation Results

**Figure 10:**
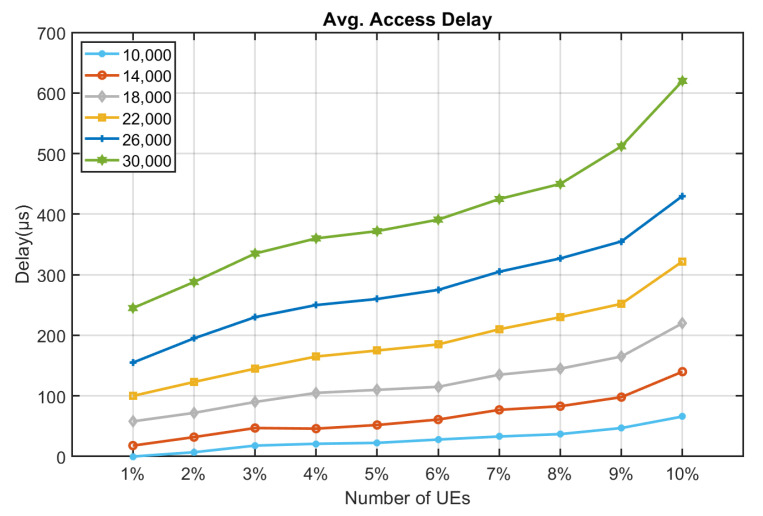
Average delay time for CE0 to send bursty messages (RU = 1).

**Figure 11:**
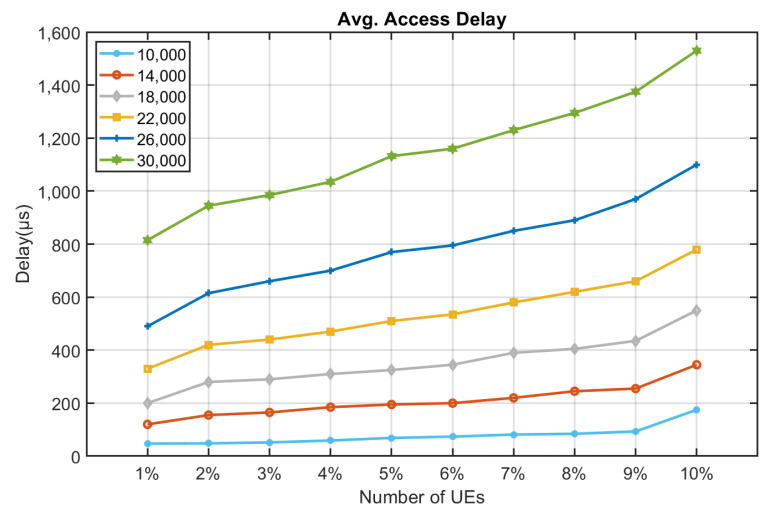
Average delay time for CE0 to send bursty messages (RU = 2).

**Figure 12:**
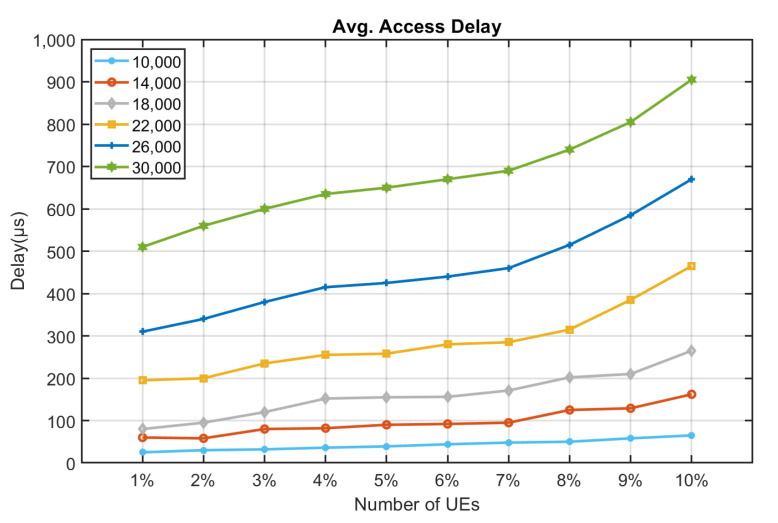
Average delay time for CE1 to send bursty messages (RU = 1).

**Figure 13:**
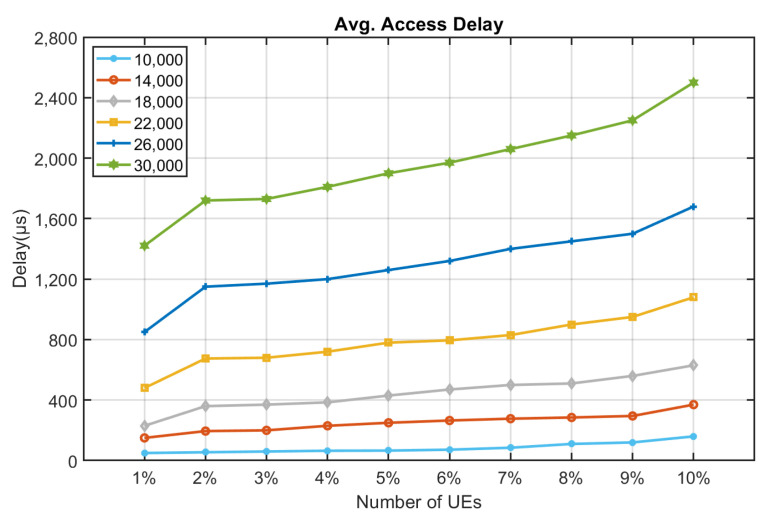
Average delay time for CE1 to send bursty messages (RU = 2).

**Figure 14:**
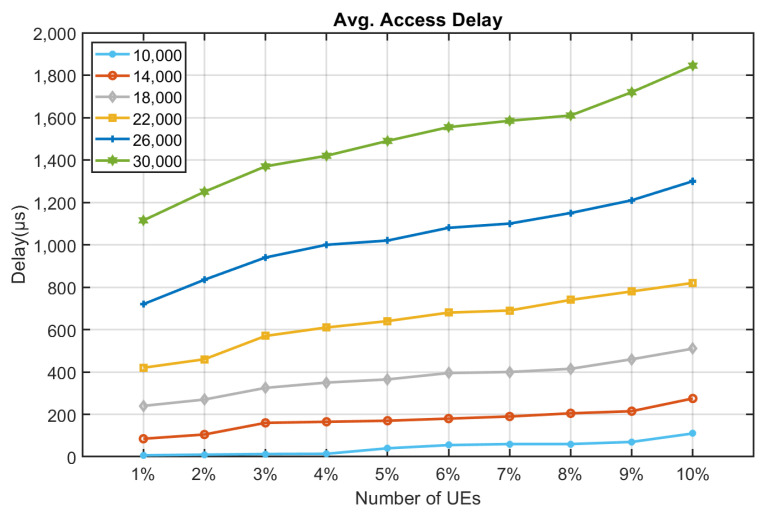
Average delay time for CE2 to send bursty messages (RU = 1).

**Figure 15:**
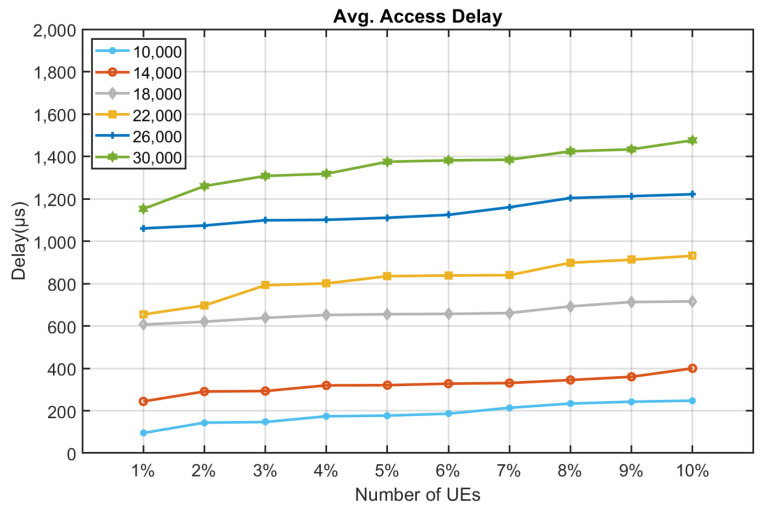
Average delay time for CE2 to send bursty messages (RU = 2).

Figure 10, Figure 11, Figure 12, Figure 13, Figure 14 and Figure 15 shows the relationship between the number of RUs required for bursty messages and the average access delay. One can observe that as the number of UE increases, the average access delay also increases. In Figure 10 and Figure 11, these access delays are very short. This means that when a bursty message needs transmission, it can receive service immediately. The reason is that slots are reserved for bursty messages. When a bursty message appears, there will be an empty slot nearby.

In Figure 12 and Figure 13, we observe that the average access delay at CE1 is longer than that at CE0. The required number of RUs for uplink transmission at CE1 is more than that at CE0, which consumes more empty slots that are reserved. So, when a bursty message needs service, it takes a longer time. Similarly, the number of RUs required at CE2 for uplink transmission is much more than CE0 and CE1. The access delay at CE2 is longer, as shown in Figure 14 and Figure 15.

**Figure 16:**
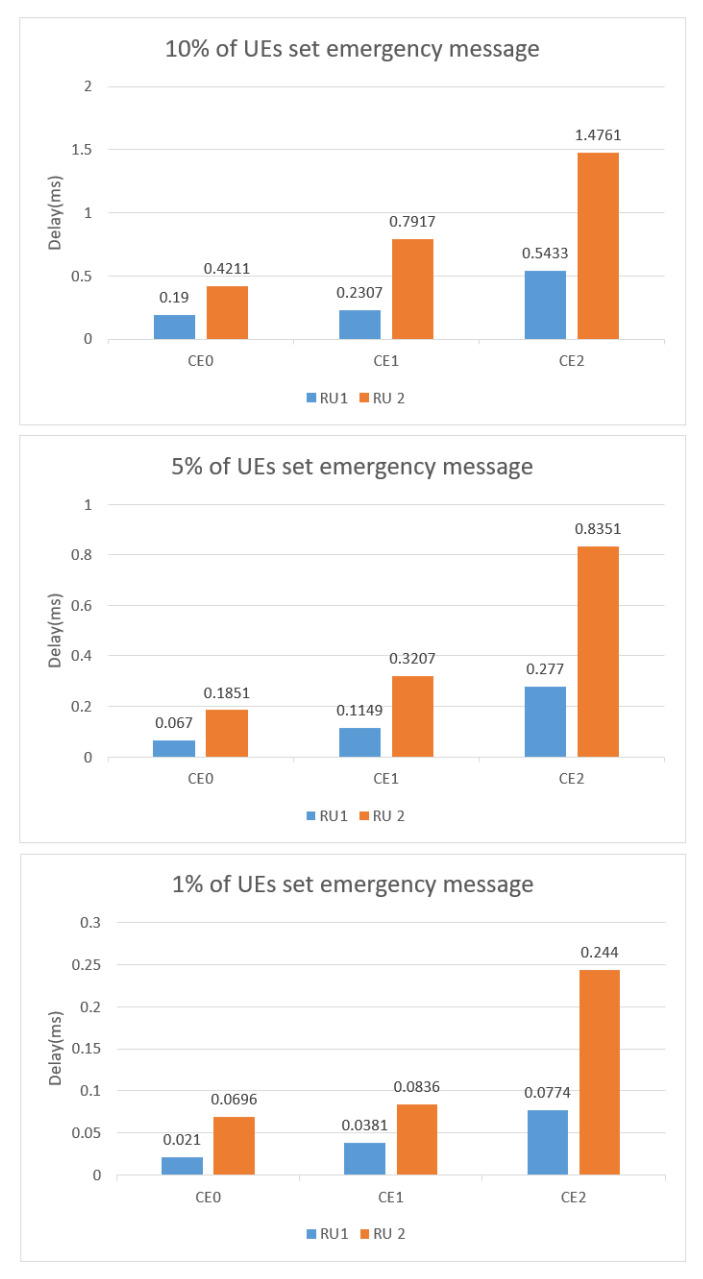
Average access delay when different % of UE sent bursty messages.

**Figure 17:**
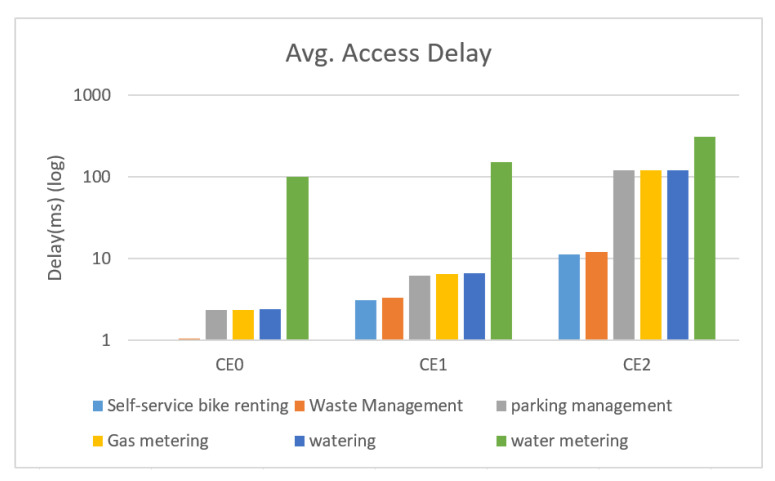
Average access delay of different devices, 10% of UE sent bursty messages (y-axis takes $log_{10}$).

Figure 16 shows the relationship between different proportions of bursty messages and delays at the three CE levels. Figure 17 shows that the number of RUs required for sending bursty messages roughly equals the number required for the UE periodic transmission. In summary, as the CE level gets higher, the bursty messages take a longer time to receive service. Moreover, as the UE increases, the waiting time to receive service also increases.

**Figure 18:**
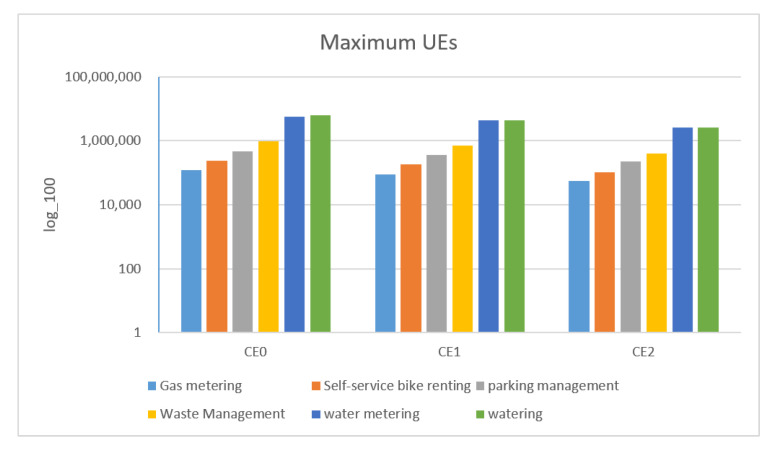
Maximum number of UEs accommodated in the system (y-axis takes $log_{100}$).

Figure 18 shows the maximum number of UE that the system can accommodate at different CE levels among devices. It reveals that when the CE level increases, the maximum number of UE that the system can accommodate decreases. The higher the CE level is, the more RUs that UE need for uplink transmissions. In other words, in a fixed period of time, the number of available slots decrease.

**Figure 19:**
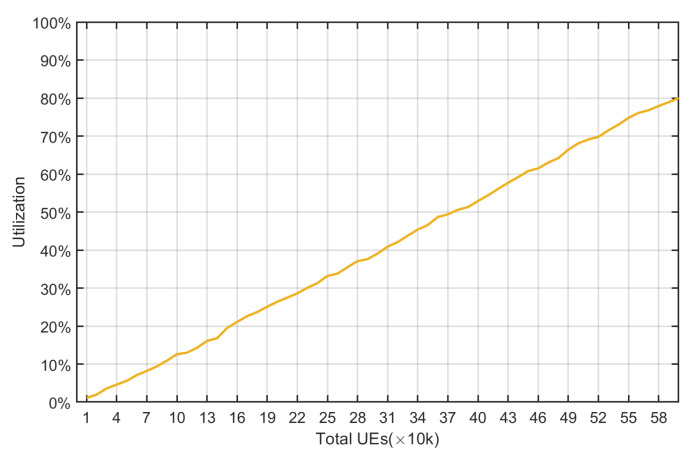
Uplink resource utilization under different number of UE.

**Figure 20:**
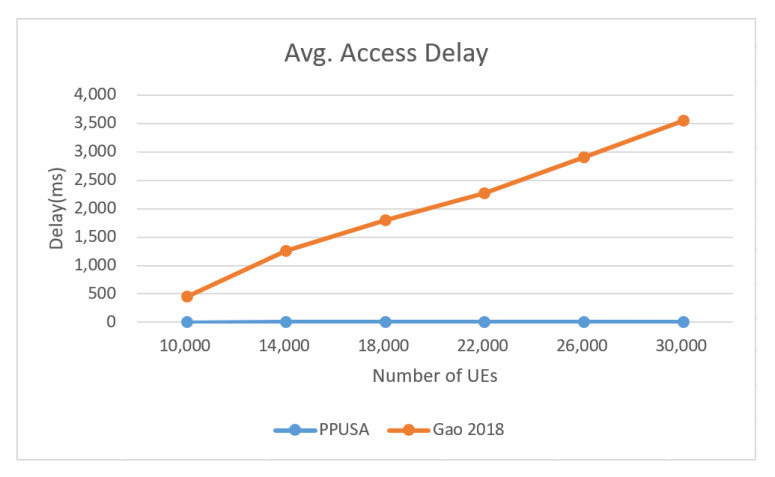
Average access delay—PPUSA and (Gao 2018).

In Figure 19, all of the different types of UE are scheduled (18 types of UE) at the three CE levels, and an observation is made for the number of different UE and the variation in the uplink resources utilization. A total of 10,000 UE were taken to begin with, and the changes were investigated after the addition of every 10,000 UE. Figure 20 shows that as the total number of UE increases, the uplink resource utilization also increases because the resources occupied increase. When the total number of UE reaches 600,000, the uplink resource utilization rate reaches 80%.

From Figure 10, Figure 11, Figure 12, Figure 13, Figure 14, Figure 15, Figure 16, Figure 17, Figure 18 and Figure 19, we can observe the trade-off between transmission delay and uplink throughput. Firstly, UE of CE level 0 have higher transmission efficiency and, thus, higher uplink throughput, and the transmission delays are therefore less than that of higher CE levels. Secondly, there is a trade-off between the transmission delay of emergency messages and uplink utilization. In the proposed algorithm, in order to guarantee low transmission latency, two subframes of each frame are reserved for the transmission of bursty messages, which limits the uplink utilization to 80% for periodic messages. The transmission delay of emergency messages will increase if fewer subframes are reserved for emergency messages, resulting in higher uplink utilization for periodic messages.

Next, the battery lifetime of UE is estimated based on the report periods and CE-levels, as shown in Table 9. Intuitively, the longer the reporting period is, the longer the battery lifetime is.

Next, a comparison is made between the proposed approach in this paper and that adopted in [24]. In [24], its RU for periodic reports is 6 tones, and its RU for bursty reports is 12 tones. In terms of delay, it was found that with the proposed approach, the average access delay required to send bursty messages was much shorter than that adopted in [24], as shown in Figure 18. Moreover, the work did not reserve slots for bursty messages, making the latency much longer than PPUSA.

In terms of battery life, the work [24] did not consider the power saving mechanism, but in the proposed approach, PSM is used to save power. As shown in Table 10, the battery lifetime of PPUSA is two-times more than [24] because the authors of [24] did not consider *RRC suspend* and *RRC resume* in their system model; the battery life of [24] is therefore lower than the proposed approach.

**Figure 21:**
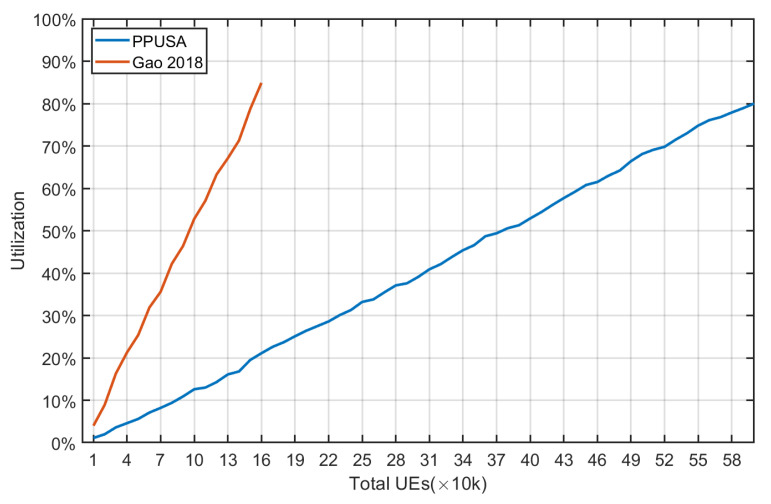
Uplink resource utilization comparison with (Gao 2018).

Finally, Figure 21 compares the uplink resource utilization of the proposed approach and [24]. It is found that the curve of [24] rises sharply and the uplink resource utilization reaches 80% when the total number of UE is 160,000. In [24], 6-tone transmission is used, occupying more resources than the proposed approach (12 tones). The results reveal that the proposed PPUSA outperforms the scheduling method presented in [24].

## 5. Conclusions and Future Works

In this paper, NB-IoT uplink scheduling for a massive number of UE was investigated and studied. In the proposed PPUSA, UE was divided into three categories: heavy, normal, and light type, respectively, based on the UE report period. The number of RUs occupied by each type of UE was also different at the three CE levels. The proposed PPUSA considers all the effects and can be used to schedule all kinds of UE. Simulation studies were used to evaluate the proposed solution. IoT devices with power-saving mechanisms were used to observe the impact on battery lifetimes under different parameter configurations. The simulation results reveal that by using PPUSA and PSM, the average access delay time, uplink resource utilization, and battery lifetime significantly improves.

In the future, we will perform a rigorous theoretical analysis of the proposed mechanism. Specifically, we will investigate more advanced topics, such as schedulability analysis. In addition, more uplink channel resources can be considered to improve uplink scheduling optimization. With the advent of 5G, researchers can also focus on NB-IoT scheduling problems for 5G networks and services. In some recent works, for example in [38], the researchers attempted to present the performance evaluation of NB-IoT in 5G heterogeneous network (HetNet) scenarios for diverse deployment strategies. Although their work was not 100% successful, they showed that, with specific techniques and approaches, NB-IoT could be helpful in a variety of services and applications for many different kinds of UE. Researchers can extend this work, and the work done by others to make NB-IoT relevant in the present and upcoming 5G HetNet use cases. Specifically, 5G and NB-IoT have applications in diverse commercial domains, such as smart cities, smart metering, smart agriculture, smart logistics, smart manufacturing, and smart homes. The combination of NB-IoT and 5G technology will play an important role in massive machine-type communications (mMTC). Moreover, 5G-narrowband IoT technologies offer exceptional advantages to consumers, enabling them to lead smarter lives [39,40].

## Figures and Tables

**Table 1:** Technical Parameters NB-IoT.

Parameters	Technical Features
Frequency	Licensed LTE frequency
Bandwidth	180 kHz
Modulation	QPSK
Multiple access	DL: OFDMA; UL: SC-FDMA
Maximum data rate	DL: 250 kbps; UL: 200 kbps
Maximum link budget	164 dBm
Bidirectional	Yes/half duplex FDD
Maximum payload length	1600 bytes
Maximum messages per day	Unlimited
Authentication and encryption	Yes (LTE encryption)
Handover	No Handover in dedicated mode

**Table 2:** Comparison of related works.

Related Works	Single Tone or Multi-Tone	Scheduling a Massive Number of UE	Support for Periodic Transmission and Bursty Transmission	CE Level	Power Saving Mechanism
[20,21]	Not Supported	NO	NO	NO	NO
[22]	Not Mentioned	NO	NO	YES	NO
[23]	Single	NO	NO	YES	NO
[15]	Single	YES	NO	YES	NO
[16]	Single	NO	NO	NO	YES
[17]	Multi	YES	YES	NO	NO
[10]	Multi	YES	NO	YES	NO
[24]	Multi	YES	YES	YES	NO
PPUSA	Multi	YES	YES	YES	YES

**Table 3:** Types of data transmission.

Type	Data Type	Characteristic
Periodic Type	Periodic Data	No requirement for delay and low need for reliability
Bursty Type	Emergency Data	High requirements for delay and reliability of data transmissions

**Table 4:** System parameters.

Parameter	Description
*T*	Simulation time (number of slots)
$N_{Heavy}$	Number of heavy type UE
$N_{Normal}$	Number of normal type UE
$N_{Light}$	Number of light type UE
$T_{Heavy}$	Report Period for heavy type UE
$T_{Normal}$	Report Period for normal type UE
$T_{Light}$	Report Period for light type UE
$T_{New}$	Report Period for newly arrived UE
$RU_{a}$	Number of RUs allocated to the UE
$RU_{New}$	Number of RUs allocated to newly-arrived UE
*id($a_{i}$)*	ID given to a specific UE
*S*	The Set of all the UE after scheduling

**Table 5:** Amount of data that one RU can carry.

	Carried Data (bit per RU)				
Efficiency	0.2344	0.377	0.6016	0.877	1.1758
1 tone (8 ms)	26.2528	42.224	67.3792	98.224	131.6896
3 tone (4 ms)	39.3792	63.336	101.0688	147.336	197.5344
6 tone (2 ms)	39.3792	63.336	101.0688	147.336	197.5344
12 tone (1 ms)	39.3792	63.336	101.0688	147.336	197.5344

**Table 6:** Simulation scenario.

Total Number of UE	10,000	14,000	18,000	22,000	26,000	30,000
Number of UE Sending bursty messages	1% to 10%					
Number of required RUs	1 or 2					

**Table 7:** Simulation parameters.

Type	Use Case	Transmission Rate	Required RUs at CE0	Required RUs at CE1	Required RUs at CE2
Light	Water metering	200 bytes/day	11 RUs	16 RUs	26 RUs
	Watering	100 bytes/12 h	6 RUs	8 RUs	13 RUs
Normal	Waste management	50 bytes/1 h	3 RUs	4 RUs	7 RUs
	Parking management	100 bytes/1 h	6 RUs	8 RUs	13 RUs
Heavy	Self-service Bike renting	50 bytes/15 min	3 RUs	4 RUs	7 RUs
	Gas metering	100 bytes/15 min	6 RUs	8 RUs	13 RUs

**Table 8:** Power Parameter Settings.

	Parameter	CE0	CE1	CE2
Power Control	Pmax	23 dBm	23 dBm	23 dBm
	RSRP	−110 dBm/15 kHz	−120 dBm/15 kHz	−130 dBm/15 kHz
	α	1	1	1
	$P0_{nominal}$	−67 dBm	−77 dBm	−87 dBm
	$P0_{UEpecific}$	0 dBm	0 dBm	0 dBm

**Table 9:** Battery life estimation.

Use Case	Report Cycle	CE0	CE1	CE2
Water metering	24 h	8.93 years	6.48 years	4.25 years
Watering	12 h	8.33 years	6.48 years	4.25 years
Waste management	1 h	1.65 years	1.25 years	264 days
Parking management	1 h	307 days	232 days	144 days
Self-service bike renting	15 min	155 days	117 days	69 days
Gas metering	15 min	78 days	59 days	37 days

**Table 10:** Battery life estimation comparison at CE0.

Use Case	PPUSA	[24]
Water metering	8.93 years	4.93 years
**Watering**	8.33 years	4.56 years
Waste management	1.65 years	307 days
Parking management	307 days	154 days
Self-service bike renting	155 days	78 days
Gas Metering	78 days	40 days

## Data Availability

Not applicable.

## Abbreviations

Some of the major topical abbreviations used in the paper are as follows:

IoT	Internet of Things
IIoT	industrial Internet of Things
NB-IoT	narrow band Internet of Things
5G	fifth generation networks
2G GSM	second generation global system of mobile communications
PPUSA	persistent periodic uplink scheduling algorithm
PSM	power saving mechanism
PBOMP	periodic block orthogonal matching pursuit
PBSBL	periodic block sparse Bayesian learning
CE	coverage enhancement
eDRX	extended discontinuous reception
3GPP	third generation partnership project
LPWAN	low power wide area networks
4G LTE	fourth generation long term evolution networks
OFDMA	orthogonal frequency division multiple access
FDMA	frequency division multiple access
SC-FDMA	single-carrier frequency division multiple access
UE	user equipment
UAD	user activity detection
TA	timing advance
RUs	resource units
NPUSCH	narrowband physical uplink shared channel
NPRACH	NB-IoT physical random access channel
RSRP	reference signal received power
SIB-NB	system information block-narrow band
MIB-NB	master information block-narrow band
DCI	downlink control information
RNTI	radio network temporary information
CRC	cyclic redundancy check
SPS	semi-persistent scheduling
VoIP	voice over internet protocol
NBPSS	narrow band primary synchronization signal
NSSSS	narrow band secondary synchronization signal
HetNet	heterogeneous network
